# Supplementary material for: Avian Metapneumovirus Subgroup C Induces Mitochondrial Antiviral Signaling Protein Degradation through the Ubiquitin-Proteasome Pathway
Source: Viruses. 2021 Oct 4;13(10):1990. doi: 10.3390/v13101990 (PMC8537000; doi:10.3390/v13101990)
Supplement: Supplementary file 1 [file viruses-13-01990-s001.zip › viruses-1398971-supplementary.pdf]

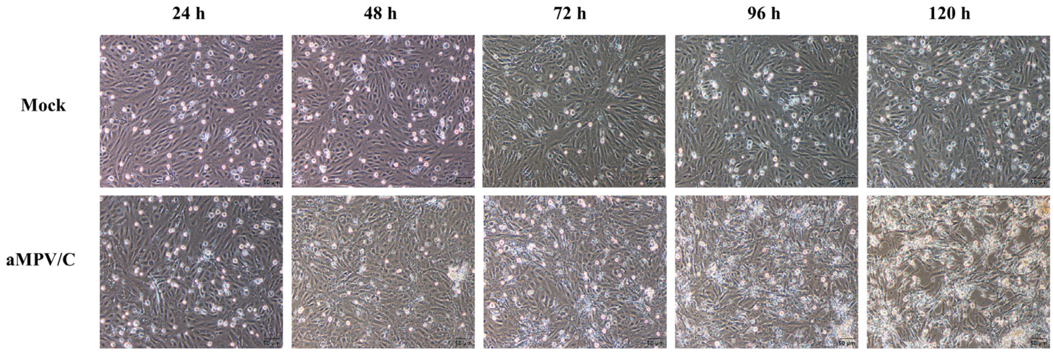

Figure S1. aMPV/C infection in Vero cells. The cytopathic effects (CPEs) caused by aMPV/C in Vero cells and mock-infected cells at 24, 48, 72, 96 and 120 h. Scale bars: 50 μm.
